# Study on Road Performance of Polyurethane Cold-Recycled Mixture

**DOI:** 10.3390/polym15081958

**Published:** 2023-04-20

**Authors:** Zhenxia Li, Tengteng Guo, Yuanzhao Chen, Tong Zhang, Deqing Tang, Menghui Hao, Xu Zhao, Jinyuan Liu

**Affiliations:** 1 School of Civil Engineering and Communication, North China University of Water Resources and Electric Power, Zhengzhou 450045, China; 2 Henan Province Engineering Technology Research Center of Environment Friendly and High-Performance Pavement Materials, Zhengzhou 450045, China; 3 Zhengzhou City Key Laboratory of Environmentally Friendly High Performance Road and Bridge Materials, Zhengzhou 450045, China; 4 Henan Communications Planning & Design Institute Co., Ltd., Zhengzhou 450046, China

**Keywords:** cold-recycled mixture, polyurethane, mix proportion design, road performance, failure mechanism, RAP

## Abstract

To give full play to the advantages of polyurethane as a binder, such as mixing at room temperature, short curing time, and high curing strength, polyurethane was used as the binder of a waste asphalt mixture, and the pavement performance of PCRM (Polyurethane Cold-Recycled Mixture) was analyzed. Firstly, the adhesion performance of polyurethane binder with new and old aggregates was evaluated using the adhesion test. Then, the mix proportion was designed according to the material characteristics, and the reasonable molding process, maintenance conditions, design indexes, and the optimal binder ratio were proposed. Secondly, the high-temperature stability, low-temperature crack resistance, water stability, and compressive resilient modulus of the mixture were evaluated through laboratory tests. Finally, the pore structure and microscopic morphology of polyurethane cold-recycled mixture were analyzed by industrial CT (Computerized Tomography) scanning, and the failure mechanism of polyurethane cold-recycled mixture was revealed. The test results show that the adhesion between polyurethane and RAP (Reclaimed Asphalt Pavement) is good, and the splitting strength of the mixture increases greatly when the ratio of glue to stone reaches 9%. Polyurethane binder has low sensitivity to temperature and poor water stability. With the increase of RAP content, the high-temperature stability, low-temperature crack resistance, and compressive resilient modulus of PCRM showed a decreasing trend. When the RAP content was less than 40%, the freeze–thaw splitting strength ratio of the mixture was improved. After the incorporation of RAP, the interface was more complex and there were many micron-scale holes, cracks, and other defects; after high-temperature immersion, the polyurethane binder appeared to show a certain degree of peeling at the holes of the RAP surface. After freeze–thaw, the polyurethane binder on the surface of the mixture produced many cracks. The study of polyurethane cold-recycled mixture is of great significance to realize green construction.

## 1. Introduction

At present, road traffic development is changing from construction to construction maintenance worldwide [1]. To achieve the national carbon peak and carbon neutralization goals, the recycling technology of old pavement materials should be actively applied [2]. How to effectively use reclaimed asphalt pavement (RAP) for asphalt pavement maintenance is of great significance to promote the development of road maintenance in resource saving [3]. Cold recycling technology has the advantages of environmental protection, high utilization, good economy, and simple construction technology [4]. Due to its technical characteristics, the traditional cold-recycled pavement method has narrow application scope and limited promotion [5]. Polyurethane is the general name for polymers containing repeat carbamate groups (–NHCOO–) on the main chain, which is formed by the addition polymerization of an organic diisocyanate or Poly isocyanate with dihydroxy or polyhydroxy compounds (polyols) [6]. Polyurethane mixture is a mixture of mineral materials with a certain gradation and a certain proportion of road polyurethane binder mixed at room temperature [7]. In recent years, polyurethane binder as a new binder has been gradually studied in the field of road engineering [8,9,10]. The adhesive property between polyurethane binder and sand is excellent [11], which can be cured at room temperature without heating and has high strength after curing. Polyurethane mixture is mainly used in high-demand pavements, such as steel bridge decks, high-grade highways, and airport runways in China [12,13,14]; by using polyurethane binder as a binder of cold recycling material, it can prolong road service life, shorten construction time and reduce operation cost, protect the environment, and reduce carbon emissions [15]. Therefore, polyurethane cold-recycled mixture (PCRM) has important research value.

Polyurethane mixture is used as a cold patch material to repair pavement defects; it has been found that polyurethane has a short curing time and high strength in practical applications, and it is feasible for use on roads [16]. According to the gradation of the asphalt mixture, a polyurethane mixture can be prepared. The thermal conductivity of polyurethane binder is much larger than that of asphalt, and the use of polyurethane binder on the road can effectively reduce road icing time [10]. Polyurethane binder has better comprehensive performance than the existing epoxy binder, which can significantly improve the high-temperature performance and flexibility of the mixture [17]. The strength of polyurethane-modified asphalt mixture is formed by the chemical reaction of isocyanate in polyurethane binder with polyol and asphalt, respectively, and it has been found that the high-temperature stability anti-ultraviolet aging of polyurethane-modified asphalt mixture is higher than those of SBS-modified asphalt mixture [18]. Polyurethane-modified asphalt mixture is essentially based on improving asphalt performance, but this cannot fill the defects of asphalt material itself. Polyurethane mixture has excellent high- and low-temperature performance and can be mixed at room temperature, which can effectively reduce energy loss and carbon emissions. It is a kind of sustainable excellent material and can completely replace asphalt to bond aggregate [19,20].

Polyurethane concrete is used to rapidly repair the surface of high-speed pavement, and it was found that polyurethane materials had rapid formation strength and good mechanical properties. Based on the advantages of the short curing time of polyurethane, polyurethane materials have been applied to the surface and base of airport runways [13]. Polyurethane asphalt concrete pavement material has good high- and low-temperature performance, low sensitivity to temperature, dynamic stability, and its maximum bending strain is about 10 times the current optimal asphalt mixture performance [21,22]. The stability of polyurethane mixture at high temperature is much higher than that of asphalt mixture, and the Marshall design method does not play a good role in controlling the performance of the mixture [23]. Polyurethane mixture is a thermosetting material, and it is not suitable to use Marshall stability and saturation as design indexes. Many design methods were introduced by American transport departments and the American Highway Transport Association, which stipulate the molding process, curing time, and design index of the recycled mixture. The specification for pavement material recycling technology in China “Technical specification for highway asphalt pavement recycling” (JTG F41-2019) [24] makes specific provisions on the test method and design index of the structural layer of the old asphalt pavement, and puts forward corresponding provisions on obtaining representative old pavement materials. Hu et al. suggested that the control indexes of the cold-recycled mixture were freeze–thaw splitting strength ratio and dry–wet splitting strength ratio, and the porosity and Marshall stability were selected as references [25]. 

To sum up, most of the current research on polyurethane binders is used to bond natural aggregates, and few scholars apply polyurethane to the field of waste pavement material regeneration. As a new type of binder, polyurethane binder has not formed a set of standard mix design method. The purpose of this study is to systematically analyze polyurethane cold-recycled mixture (PCRM). Starting from the characteristics of raw materials, the mixed design method is explored. Through many indoor tests, the road performance of PCRM under different RAP content is verified, and the microscopic characteristics of the mixture are analyzed in depth by micro means. The main research contents include raw material performance analysis, mix proportion design, road performance research, and micro-mechanism analysis. At present, the use of waste pavement materials needs to be solved urgently. This paper proposes to use polyurethane binder as an RAP binder and tries to use new ways to strengthen the utilization of waste pavement materials. The research on polyurethane cold-recycled mixture helps to reduce the cost of pavement maintenance, reduce the exploitation of natural stone raw materials, reduce carbon emissions, and achieve green construction.

## 2. Materials and Methods

**Figure 1:**
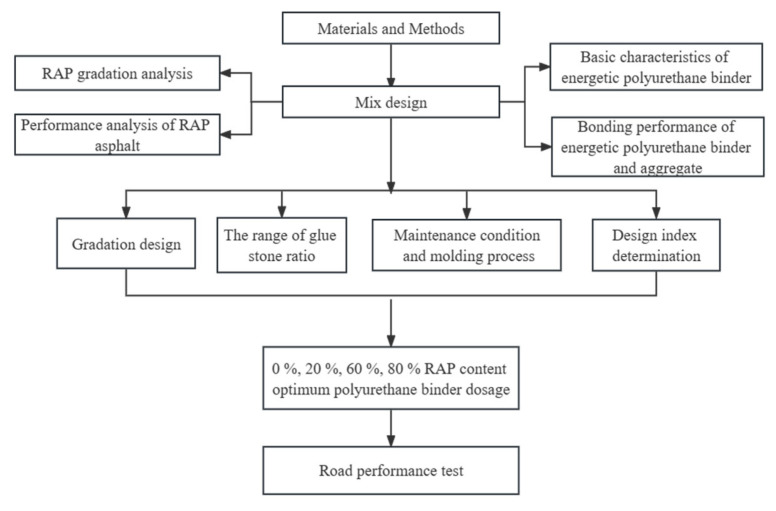
Flowchart of materials and methods.

The raw materials of PCRM mainly include RAP, polyurethane binder, and natural aggregates added to adjust the gradation of the mixture. To avoid the influence of RAP material variability on mixture performance, it is necessary to analyze the RAP variability used in this test. At the same time, due to the different synthetic materials and synthesis processes, there are many types of polyurethane binder, and their basic properties should be tested. Therefore, this chapter will carry out PCRM raw material performance tests to fully understand the material properties. The flowchart is shown in Figure 1.

### 2.1. RAP

The recycled asphalt mixture samples used in the test are all the upper layers of asphalt concrete pavement, which were obtained by milling with a Vintage cold milling machine after the extraction and screening tests in the laboratory. The performance test was carried out according to the specification of [26].

#### 2.1.1. Gradation of RAP

**Figure 2:**
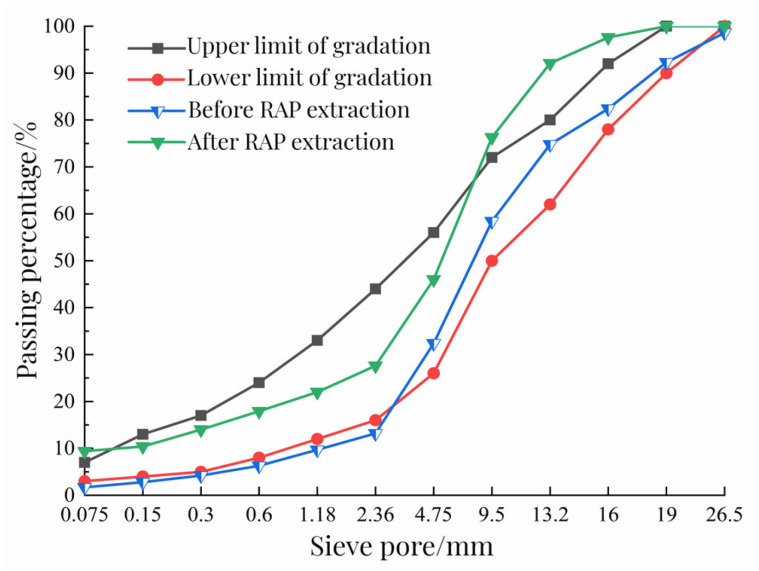
Aggregate gradation before and after RAP extraction.

To explore the change rule of RAP before and after extraction, this test used an LLC-30 automatic asphalt mixture extractor to extract RAP. The instrument is produced by Cangzhou Kexing Equipment Co., Ltd. (Hebei, China). First, 1200 g of material was weighed by the quartering method. After the screening test, it was placed in a beaker containing trichloroethylene. When RAP was fully dissolved, the mixture and solution were poured into the extractor. Next, the weighed filter paper was placed on the edge of the instrument, and the centrifuge was opened, and new solvent was continuously added for washing. After the outflow liquid became pale yellow, the extraction was ended. After the filter paper was dried in the oven, the increased mass was weighed as the quality of the mineral powder attached to the top. After multiple extractions, the whole ore was put into the sieve shaker for the screening test. The screening results are shown in Table 1 and the RAP grading curve before and after extraction is shown in Figure 2.

It can be seen from Figure 2 that the maximum particle size of RAP in advance is 26.5 mm, and the maximum particle size after extraction is 19.0 mm; the particle size content below 2.36 mm in RAP before extraction does not meet the requirements of the specification lower limit, which is caused by the “refinement” phenomenon of RAP material. On the one hand, RAP breaks the aggregate under the action of the milling cutter in the milling process, resulting in the refinement of RAP, reducing the content of large particles, and increasing the variability of RAP materials. On the other hand, the aggregate is crushed because of the long-term traffic load on the road, resulting in increased fine material content. After centrifugal extraction, the passing percentage of raw materials above 9.5 mm in RAP has exceeded the upper limit of grading; compared with the passing percentage of 0.75 mm particle size before and after extraction, the passing percentage after extraction was significantly greater than that before extraction, indicating that the powder increased after RAP extraction. The reason is due to the “agglomeration” effect of RAP. After planning and milling, large-particle RAP is not completely coarse aggregate, but consists of many smaller fine particles and asphalt blocks. From the screening results, the agglomeration effect of RAP is obvious at the particle size of 26.5 mm. The above results show that the variability of RAP is large. To avoid the “refinement” and “agglomeration” effect of RAP on the performance of the mixture, the RAP material was screened and classified into 0~5 mm, 5~10 mm, and 10~20 mm three-grade aggregates, and the three-grade RAP material was screened and tested to determine its gradation composition. The results of RAP classification are shown in Table 1.

#### 2.1.2. RAP Asphalt

The aged asphalt in the solution obtained after the extraction test was recovered by referring to the rotary evaporator method. The instrument used in the experiment was the LHS-205D rotary evaporator, which is produced by Beijing Blue Aviation Science and Technology Co., Ltd. (Beijing, China). The asphalt solution was obtained after the extraction test was collected, and the aggregate in the sieve was poured out, with 70# matrix asphalt as the control group, the basic performance of aged asphalt in RAP after multiple recoveries was tested, and the test results are shown in Table 2.

It can be seen from Table 2 that the asphalt in RAP has been seriously aged. Compared with the performance of asphalt, the penetration degree and prolongation of aged asphalt in RAP have been significantly reduced, and the softening point and viscosity have been increased. Based on the aging mechanism of asphalt, the components of asphalt will change after aging; due to the decrease of the dispersion medium, the solubility of hydrocarbon compounds is weakened, resulting in the decrease of resin and the increase of asphaltene content. At the same time, after the oxidation reaction of asphalt, the molecules polymerized into a network structure, and the structure of asphalt changed from soluble gel type to gel type. At the macro level, the viscosity increased, and the liquidity decreased. Therefore, after long-term service of asphalt pavement, affected by vehicle load, natural conditions, and other factors, the asphalt in RAP has been seriously aged, and its technical performance has been significantly reduced. RAP can be seen as a “black” aggregate.

### 2.2. Natural Aggregate

In this paper, the addition of new aggregates and fillers was mainly used to optimize the gradation of the mixture and play the role of skeleton embedding. The coarse and fine aggregates with good angularity, clean surface, and particle size of 0~5 mm, 5~10 mm, and 10~20 mm were selected, and lava rock mineral powder was used as filler. The properties of coarse and fine aggregates and mineral powder are in line with the performance requirements of raw materials in the asphalt pavement specification of China [27]. Hygroscopic rate refers to the property that the material absorbs water in contact with water, and crushing value refers to the performance index of aggregate resistance to crushing. The performance test results and screening results of the aggregates are shown in Table 3 and Table 4.

### 2.3. Energetic Polyurethane Binder

#### 2.3.1. Basic Characteristic

The two-component polyurethane used consists of polyol (component A) and isocyanate (component B); when used, the A and B components are mixed according to the mass ratio of 5:1. After full stirring, the hydroxyl group on the polyol will react with the isocyanate group on the isocyanate, and then cross-link and solidify. Referring to the test methods of relevant specifications [28,29,30,31], the performance test results of the two-component polyurethane adhesive used in this paper are shown in Table 5.

#### 2.3.2. Evaluation of Adhesive Property

**Figure 3:**
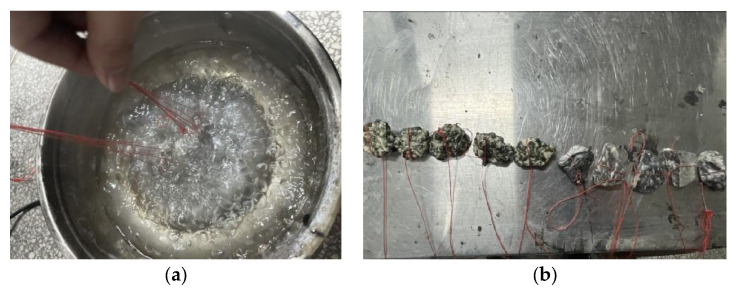
Polyurethane binder and aggregate adhesion test. (**a**) In boiling; (**b**) after boiling.

The initial state of polyurethane binder is liquid. When it penetrates the aggregate, the two components of polyurethane binder can be cured by chemical reaction, which can well anchor the aggregate and increase the mechanical adhesion between aggregates. The adhesion mechanism of this process is overly complex, including chemical reaction and physical reaction, and there is also a selective diffusion effect of polyurethane binder. Due to the limitation of test conditions, there is no condition to directly determine the bonding strength of polyurethane binder, so this paper uses indirect tests to analyze. The film of asphalt adhering to the surface of coarse aggregate was peeled off by water at a certain temperature to judge the adhesion performance of asphalt and aggregate surface. According to the adhesion test of emulsified asphalt and coarse aggregate in the specification of [26], the adhesion performance of polyurethane binder with natural aggregate and RAP material was evaluated. The test results are shown in Figure 3.

It can be seen from Figure 3 that after boiling for 3 min, the coating area of polyurethane binder with natural aggregate and RAP was more than two-thirds, which meets the requirements of the specification, indicating that polyurethane binder has good adhesion performance with natural aggregate and RAP.

### 2.4. Composition Design of Gradation

**Figure 4:**
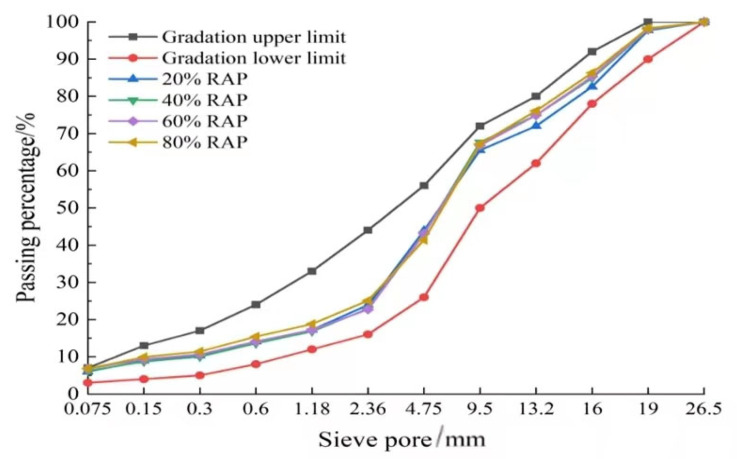
Four kinds of RAP mixing level distribution curves.

In this paper, AC-20 asphalt mixture gradation was selected for PCRM. Combined with the screening results of natural aggregate and RAP in Chapter 2, the proportion of natural aggregate and RAP was adjusted to make it as close to the median of gradation as possible; a total of PCRM gradations with four RAP dosages of 20%, 40%, 60%, and 80% were designed. The gradation curves of four RAP dosages are shown in Figure 4.

### 2.5. The Range of Binder Ratio

After fully mixing with the aggregate, the polyurethane adhesive can form a uniform mortar layer on the surface of the aggregate. When the binder ratio is small, the polyurethane adhesive cannot completely wrap the aggregate, which will lead to poor bonding performance between the aggregates. When the binder ratio is large, a certain amount of polyurethane binder will remain at the bottom of the mixture, resulting in the phenomenon of mortar seal. After mixing the mixture with different binder ratios, it can be clearly observed that the polyurethane mixture has different mixing states.

**Figure 5:**
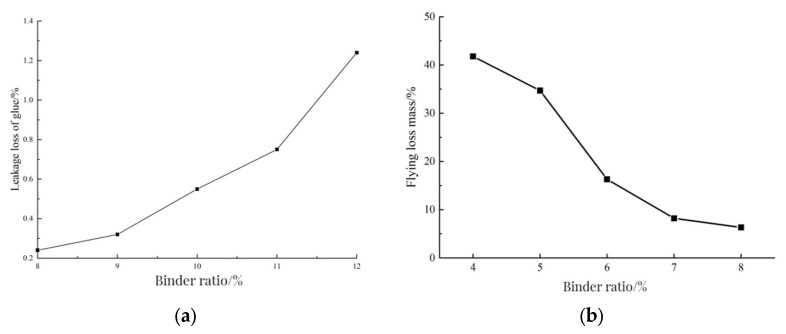
Scattering and leakage test results. (**a**) Influence of cement–stone ratio leakage loss. (**b**) Results of scattering experiment.

The Cantabro test can reflect the anti-loosening performance of the mixture. The minimum binder ratio of PCRM is determined by the Cantabro test. The binder ratios of 4%, 5%, 6%, 7%, and 8% were selected to make standard Marshall specimens. After 2 days of curing at room temperature, the mass loss of the specimens was measured after rotating for 300 revolutions in the Los Angeles abrasion tester. The number of specimens in each group was 4, and the average value was taken as the test result. The maximum binder ratio of PCRM was determined by referring to the Schellenberg leakage test. Firstly, the binder ratios of 12%, 11%, 10%, 9%, and 8% were selected according to the state of the mixture during the trial mixing. Then, after the polyurethane binder was fully mixed with the aggregate, each group of mixture was divided into four parts on average. The mass of each part of the mixture was weighed and poured into an 800 mL beaker, and the quality of the beaker and the mixture was weighed. Because the polyurethane adhesive had good fluidity and short curing time in the initial stage of mixing, the quality of the beaker and the adhesive attached to the beaker was weighed after standing at room temperature for 30 min. The average value of the four groups of tests was taken as the test result, and the leakage loss rate of the mixture was obtained. The relationship between the leakage loss rate and the binder ratio is shown in Figure 5a.

The range of polyurethane–binder ratio was determined by the Cantabro test and leakage test results. Figure 5b shows the relation curve between the scattering loss and the binder ratio. With the increase of the binder ratio, the mass of the dispersion loss gradually decreased; when the binder ratio was small, the dispersion loss of the specimen was large, and the bonding force between the polyurethane binder and the aggregate was insufficient; when the binder ratio reached 7–8%, the mass loss of the specimen tended to be stable, but large damage occurred inside the specimen. At this time, the mass loss of the specimen was mainly caused by the rupture of RAP when it collides with the steel ball. When the binder ratio reached 8%, the dispersion mass loss was the smallest, and the bonding strength between polyurethane binder and aggregate remained the best. The minimum binder ratio of PCRM was 8%.

Figure 5b shows the relation curve between the mass loss of leakage and the ratio of binder ratio; with the increase of the ratio of glue to stone, the loss rate of leakage of the mixture also increased, when the ratio of glue to stone increased from 8% to 10%, the loss rate of leakage of the mixture increased slowly. When the ratio of glue to stone was 10%, the loss rate of leakage of the mixture was 0.28%, which meets the requirement of no more than 0.3% in the specification of [32]; when the ratio of glue to stone increased from 10% to 12%, the loss rate of leakage of the mixture increased rapidly. This is due to the binder ratio being too large; the polyurethane binder, due to surplus resulting from the segregation phenomenon, will be in the inner wall and bottom of the adhesive agent. When the binder ratio was 11%, the leakage loss rate of the mixture exceeded the required value in the specification.

The results of the Cantabro test and leakage test show that the adhesive strength between polyurethane binder and aggregate can be maintained well and that there is no segregation phenomenon. When the binder ratio was in the range of 8–10%, combined with the mixing state of the mixture after trial mixing, the binder ratio of PCRM was determined to be 8–10%.

### 2.6. Molding Procedure

When determining the mixing process, it is mainly considered that the polyurethane binder can be cured at room temperature and uniformly cover the aggregate surface. Referring to the asphalt test procedure mixture mixing method, the binder ratio of the mixture was selected to be 9% for the test. Firstly, the mixing pot temperature was set to 25 °C. After preheating, all aggregates except mineral powder were mixed in the mixing pot for 90 s, then polyurethane binder was added to mix with aggregates for 90 s, and finally mineral powder was added. Continuing to stir for 90 s, the total mixing time of the mixture was 4.5 min, which is 1.5 min longer than the standard mixing time of asphalt mixture, so that the polyurethane binder can better wrap the aggregate.

**Figure 6:**
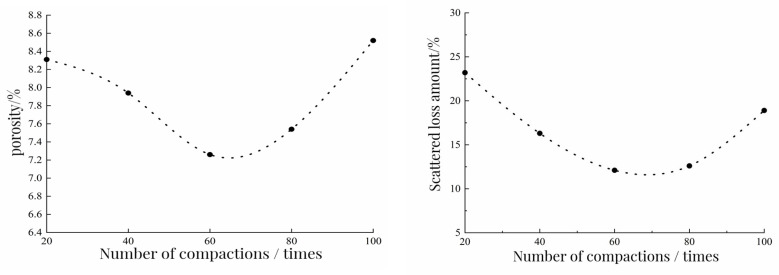
The relationship between porosity and scattered loss and compaction times.

In this test, the specimen was formed by single compaction. In the single-hit real-time, selecting the appropriate number of compactions is conducive to reducing the porosity of the mixture. In this section, 80% RAP gradation and 8% binder ratio were used to study the compaction method of the specimen. The specific research methods were: after the mixing of the mixture was completed and after standing at room temperature for 20 min, the two sides were compacted 20 times, 40 times, 60 times, 80 times, and 100 times, respectively. Taking the porosity and loss of the specimen as the index, the best compaction times were reasonably selected. The relationship between the porosity of the specimen and the amount of scattered loss after the Cantabro test and the number of compactions is shown in Figure 6.

It can be seen from Figure 6 that the scattering loss and porosity of the specimen decreased first and then increased with the increase of the number of compaction times. When the number of compaction times increased from 20 to 60, the porosity and scattering loss of the specimen gradually decreased. There was an inflection point between 60 and 80 times. After the inflection point, the porosity and scattering loss of the specimen gradually increased with the increase of the number of compaction times. After 100 compactions, it was found that the edge of the specimen had been damaged to a certain extent. The number of compactions at the inflection point was about 70 times. The compactness of the mixture under this number of compactions was higher, and the adhesion between polyurethane binder and aggregate was better. Therefore, we chose a single double-sided compaction 70 times to form the specimen.

### 2.7. Maintenance Temperature and Time

**Figure 7:**
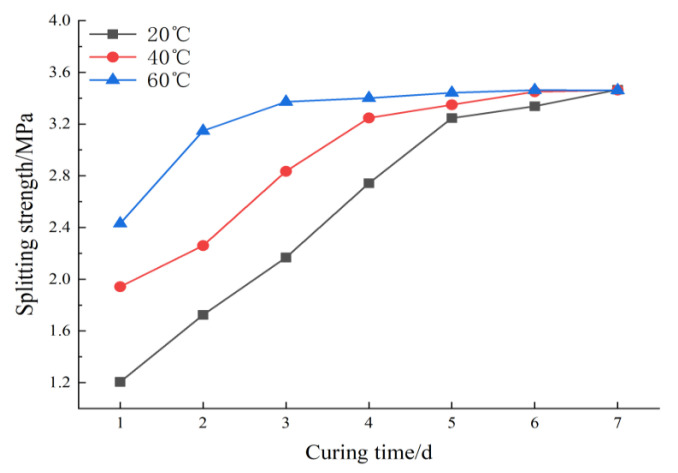
Splitting strength at different curing temperatures.

In this experiment, Marshall specimens were formed by 80% RAP dosage gradation and 8% binder ratio combined with the above molding process. The curing temperatures were 20 °C, 40 °C, and 60 °C, each group of specimens was cured for 1–7 days, and the splitting strength of each group was measured. The variation of splitting strength in specimens under different curing conditions is shown in Figure 7.

From Figure 7, it can be seen that the splitting strength at 40 °C and 60 °C maintenance temperatures are close to the maximum splitting strength earlier than that at 20 °C maintenance temperature. The specimens at 60 °C, 40 °C, and 20 °C are close to the maximum splitting strength at 2d, 4d, and 5d, respectively, and then strength is almost not increased. This shows that high temperature helps to improve the curing rate of polyurethane binder, and the strength growth rate of specimens is gradually accelerated. When the specimens were maintained for 5d–7d, the splitting strength of each group tended to be stable, and the strength increased slightly. By comparing the splitting strength of the specimen after 7 days of curing, the splitting strength of the three maintenance temperatures were almost the same and the temperature did not affect the final strength of the specimen. At the maintenance temperature of 60 °C, although the softening point of asphalt in RAP has been reached, the adhesion between polyurethane binder and aggregate was maintained well without negative impact. In conclusion, PCRM needs suitable maintenance conditions to achieve the highest strength; increasing the curing temperature can improve the curing rate and make the specimen form strength quickly.

### 2.8. Optimum Amount of Polyurethane Binder

To determine the optimal dosage of polyurethane binder, the dry–wet splitting strength ratios of four RAP dosages under different binder ratios were determined according to the method described in the regeneration specification [24]. According to the mixture forming process, two groups of standard Marshall specimens with different RAP contents were prepared, respectively. After accelerated curing in a blast drying oven at 60 °C for 2 days, 1 group of specimens was immersed in a constant-temperature water bath at 15 °C for 2 h and then taken out for splitting strength test. In the other group, the specimens were first placed in a constant-temperature water bath at 25 °C for 22 h, and then in a constant-temperature water bath at 15 °C for 2 h. The splitting strength value was measured.

It can be seen from Table 6 that the splitting strength of the mixture increases with the increase of the cement–stone ratio. Firstly, under the same RAP content, when the binder ratio of PCRM increased from 8% to 9%, the splitting strength of the specimen increased rapidly with the increase of binder ratio, and when it increased to 10%, the strength growth rate slowed down. Taking 60% RAP content as an example, when the binder ratio increased from 8% to 9%, the 15 °C splitting strength of the specimen increased by 8.6%, while when the binder ratio increased to 10%, the strength only increased by 2.7%. This is because RAP has less indirect contact surface in the mixture, and the strength of the specimen is mainly provided by the bonding force of polyurethane binder, so the splitting strength of the specimen will rise rapidly when the ratio of binder increases. Continuing to increase the binder ratio only plays a role in thickening the surface film of the aggregate, and the effect of improving the strength is not obvious. Therefore, the strength growth rate will slow down.

Secondly, with the increase of the binder ratio, the dry–wet splitting strength ratio also increased, but when the binder ratio was 8%, the dry–wet splitting strength ratio under the four RAP contents did not meet the requirements of the dry–wet splitting strength ratio greater than 75% in the regeneration specification. When the binder ratio increased to 9%, the dry–wet splitting strength ratio was greater than the specification requirements, and its water damage resistance met the use requirements. In summary, the optimal binder ratio of PCRM was determined to be 9%.

## 3. Test Scheme

### 3.1. Pavement Performance Test

When PCRM is applied to roads, the service environment is more complex, not only to bear the fatigue load of vehicles, but also because it is affected by natural factors in the actual environment. At the same time, RAP material has many defects and asphalt agglomerates on the surface, and its material stress point is relatively special, resulting in RAP content affecting the road performance of PCRM. Therefore, exploring the influence of RAP content on the road performance of PCRM is helpful to verify the design rationality of the mixture. According to the mixture ratio design of 20%, 40%, 60%, and 80% RAP content in the third chapter, the polyurethane mixture with all-natural aggregate (0% RAP content) was added as the control group. The pavement performance of PCRM was tested by referring to the asphalt mixture test procedure. The high-temperature stability, low-temperature crack resistance, water stability, and compressive elastic modulus of PCRM under different RAP content were compared and analyzed.

### 3.2. Scanning Electron Microscope

In this experiment, a Gemini Sigma 300 ultra-high resolution field emission scanning electron microscope (SEM) produced by Zeiss, Germany was used, as shown in Figure 6. The PCRM specimens were treated by simulating the high-temperature immersion conditions and freeze–thaw cycles of the mixture; the microscopic morphology of the polyurethane-aggregate interface was observed by scanning electron microscopy, and the changes of the microscopic morphology of PCRM under the coupling effect of water temperature were analyzed. There were four groups in the test plan: (1) 0% RAP dosage PCRM, (2) 60% RAP dosage PCRM, (3) PCRM with 60% RAP content after high-temperature immersion, and (4) PCRM with 60% RAP content after freeze–thaw cycles. After the end of the simulation environment, the dried sample was fixed on the sample table with conductive adhesive, and the test was carried out after spraying gold.

### 3.3. Industrial CT Test

Industrial CT is the abbreviation of industrial computer tomography technology. Compared with conventional detection technology, industrial CT can be used for non-destructive testing of samples, which can provide two-dimensional and three-dimensional images of the geometric structure of the sample, gap defects, and other information. Through industrial CT three-dimensional imaging technology, the internal defects of the material can be found quickly and intuitively, and the porosity, uniformity, and fiber orientation of the composite material can be calculated by the software.

**Figure 8:**
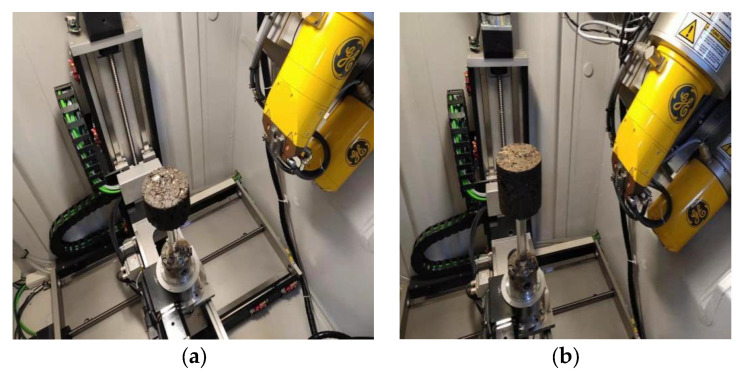
Industrial CT specimen scanning. (**a**) Marshall hammer sample. (**b**) Vibratory compactor sample.

Industrial CT has been widely used in many fields. For road materials, industrial CT is mainly used to analyze the distribution characteristics of voids and cracks in materials. For asphalt mixtures, using industrial CT technology can study the surface void ratio along the depth direction of different layers of asphalt concrete and actual pavement under different molding methods. In this experiment, industrial CT with the model of phoenixv|tome|xs was used for the test. To explore the effect of different molding methods on the void fraction of RCPM and the failure mechanism under different load forces, the molding method of the specimen was mainly the Marshall compaction method and the vibration compaction method. At the same time, the splitting strength test and uniaxial compression test of the specimen were mainly carried out. Four experimental schemes were designed: ① before the splitting failure of Marshall compaction molding, ② splitting failure after Marshall compaction, ③ before uniaxial compression failure of vibration compaction, and ④ after uniaxial compression failure of vibration compaction. The effects of different molding methods on PCRM porosity can be judged by comparing the experimental groups ① and ③. The failure mechanism under different loads can be judged by comparing the experimental groups ② and ④. After the specimen was made, it was scanned in industrial CT, as shown in Figure 8.

## 4. Results and Discussion

### 4.1. High-Temperature Stability Performance

The high-temperature stability of PCRM was evaluated by the Marshall test and rutting test. Five kinds of Marshall specimens with RAP content were prepared, with four specimens in each group. After the end of curing, the Marshall test instrument and the specimens were placed in a constant-temperature water bath at 60 °C for 30 min, and then the Marshall stability test of RCPM was carried out. The rut plate specimens with a size of 300 mm × 300 mm × 50 mm were formed by the wheel rolling method and put into a 60 °C blast drying oven to accelerate the curing for 2d. Before the test, the rut plates with 5 kinds of RAP content were placed in a 60 °C tester for 5 h, and the rolling frequency of the testing machine was selected as 42 times/min. After each specimen traveled 60 min along the surface, the test results were measured. The test results are shown in Table 7.

It can be seen from Table 7 that Marshall stability is negatively correlated with RAP dosage; when RAP dosage was large, Marshall stability of the mixture decreased greatly. This is due to the decrease of natural aggregate content with the increase of RAP content. At high temperatures, asphalt softens in RAP, the larger particle group loses cohesiveness and transforms into finer aggregate, and the mixture gradation is weakened. At the same time, less coarse aggregate cannot play a good skeleton role, and the interlocking ability between aggregates is reduced, which reduces the ability of PCRM to resist load deformation. The dynamic stability is used as the evaluation index in the rutting test; the greater the dynamic stability is, the better the high-temperature performance of the mixture is. It can be seen from Table 7 that the dynamic stability of PCRM decreases with the increase of RAP dosage and the increase of RAP dosage aggravates the rutting depth, but the deformation extent is maintained in a small range. The dynamic stability of specimens with 80% RAP dosage is the smallest, but it is much higher than that of SBS-modified asphalt mixture, about 7000 times/mm [33]. The test results show that PCRM has strong anti-rutting deformation ability, and under the action of high temperature and repeated vehicle load, the mixture almost does not produce plastic deformation after curing. Due to the viscoelasticity of aged asphalt in RAP, it is easy to creep under high temperatures, which leads to stress relaxation; the increase of RAP dosage will reduce the high-temperature stability of the mixture.

The high-temperature stability of PCRM was evaluated by the Marshall stability test and rutting test. The results showed that Marshall stability and dynamic stability decreased with the increase of RAP content. Compared with PCRM with 0% RAP content, the Marshall stability of PCRM with 20%, 40%, 60%, and 80% RAP content decreased by 6.3%, 9.1%, 13.1%, and 16.9%, respectively, and the dynamic stability decreased by 5.8%, 8.5%, 14.3%, and 21.7%, respectively. This is because with the increase of RAP content, in a high-temperature environment, the asphalt in RAP is softened, the larger particle group loses cementation and transforms into finer aggregate, the gradation of the mixture is weakened, the coarse aggregate cannot form the skeleton structure, and the PCRM resistance to load degeneration is reduced. Overall, the rut depth of the specimen after the rut test is very small, and the polyurethane binder hardly produces plastic deformation after curing to form strength. The high-temperature stability of PCRM is better.

### 4.2. Moisture Stability

The immersion Marshall test can effectively simulate the water damage resistance of the mixture under high-temperature and rainy conditions in summer. In this test, standard Marshall specimens were prepared according to the molding method mentioned above and the curing conditions. After the curing of the specimens, they were placed in a constant temperature water bath at 60 °C for 48 h. After the Marshall stability test, the immersion residual stability of the mixture was calculated. The immersion dispersion test is mainly used to evaluate the adhesion performance of the polyurethane binder and the aggregate under the immersion condition. First, the Marshall specimens after curing were subjected to a standard dispersion test, and the other group was immersed in a constant-temperature water bath at 60 °C for 48 h. After being placed at room temperature for 24 h, the dispersion test was performed to measure the mass loss of the specimens before and after immersion, which was used as the evaluation index of the water stability performance of the PCRM. The freeze–thaw splitting test was used to evaluate the water stability of the mixture by simulating the change of the splitting strength of the mixture under the action of alternating hot and cold environment. First, two groups of Marshall specimens with different RAP contents were formed. One group of specimens was vacuum-saturated, and the specimens were placed in a plastic bag. After adding 10 mL water to seal, they were frozen in a −18 °C low-temperature refrigerator for 16 h. After the freezing was completed, they were placed in a 60 °C constant-temperature water bath for 24 h. Then, the two groups of specimens were placed in a 25 °C water bath for 2 h for splitting test. At the end of the test, the ratio of splitting strength before and after freezing and thawing was calculated, and the strength ratio of freeze–thaw splitting test (TSR) was used as the evaluation index.

**Figure 9:**
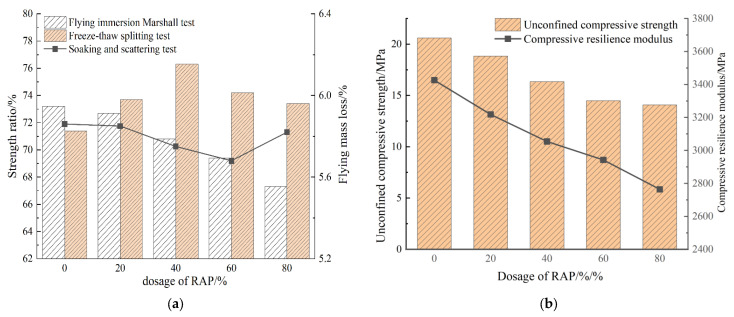

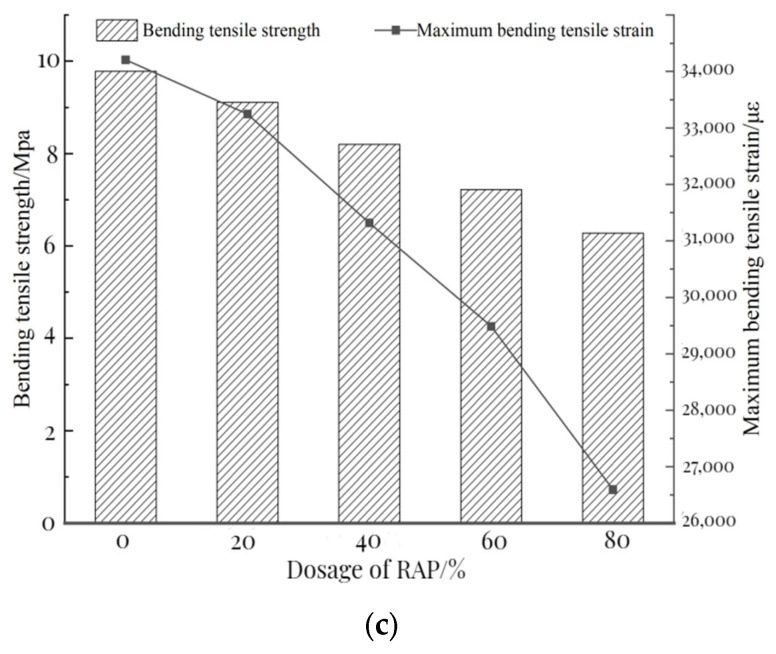
The influence of RAP content on three kinds of road performance. (**a**) Effect of RAP content on water stability. (**b**) Relationship diagram between uniaxial compression test results and RAP dosage. (**c**) Relationship between flexural strength and maximum flexural strain of PRCM and RAP dosage.

The water stability of PCRM was analyzed using the immersion Marshall test, immersion dispersion test, and freeze–thaw splitting test. By comparing the results of the three tests, it was found that when the water stability of PRCM was evaluated according to a single index, the conclusions were quite different; according to the test results, the changing trends of the three tests under different RAP dosages were plotted as shown in Figure 9a.

It can be seen from Figure 9a that in the immersion Marshall test, the residual stability of the specimen decreased with the increase of RAP dosage. In the freeze–thaw splitting experiment, the splitting strength ratio increased first and then decreased with the increase of RAP dosage. The nature of the force on the specimen in the freeze–thaw splitting test is different from that in the Marshall stability test; the main force on the specimen in the splitting test is the tensile stress generated by the external load. When the internal bonding force of the specimen is insufficient, it will lead to damage of the specimen. In the Marshall stability test, the specimen is mainly affected by radial pressure, and the main influencing factor is the friction between mineral particles, so there is a certain gap between the two.

The comprehensive effects of temperature, water and vehicle load on roads in actual road use can be effectively simulated by the above two test conditions. The immersion dispersion test is mainly used to evaluate the anti-loose performance of the mixture under the action of water; the test results are significantly different from the immersion Marshall test and the freeze–thaw splitting test as when the strength loss is large, the dispersion loss of the specimen remains small, indicating that the bond strength loss of polyurethane adhesive and aggregate is not serious, but the bond surface between polyurethane and stone material may be in a state of failure, and the internal aggregate has been damaged.

The results of the immersion Marshall test and the freeze–thaw splitting test showed that the water damage resistance of PCRM was poor. Compared with PCRM with 0% RAP content, the residual stability of the 20%, 40%, 60%, and 80% mixtures decreased by 0.5%, 2.4%, 3.8%, and 5.9%, respectively. TSR increases first and then decreases with the increase of RAP content, but it is greater than that of the mixture without RAP, which indicates that the incorporation of an appropriate amount of RAP is beneficial to improving the water damage resistance of PCRM. The results of the immersion dispersion test show that the bonding performance between polyurethane binder and aggregate remains good after immersion, and the dispersion loss can still maintain a small value when the strength decreases significantly.

### 4.3. Compressive Modulus of Resilience

The unconfined compressive strength and compressive rebound modulus of PCRM were measured according to the uniaxial compression test method in asphalt mixture. The test temperature was 20 °C. A cylindrical specimen of φ 100 mm × 100 mm was made using a vibrating compactor. After the curing of the specimen, the unconfined compressive strength was first measured by a universal material testing machine, and then the maximum value of the unconfined compressive strength was evenly distributed into 10 levels. Taking 0.1~0.7 Pa as the test load, the step-by-step loading and unloading method was adopted. The loading rate of the testing machine was 2 mm/min and was unloaded immediately after each level of load. At the same time, the difference between the displacement of the loading and unloading was recorded as the amount of rebound deformation. The variation of the test results with the RAP content is shown in Figure 9b.

It can be seen from Figure 9b that the addition of RAP has a great influence on the compressive strength and compressive resilient modulus of PCRM. The test results show that the strength and stiffness of PCRM are greatly lost when the RAP dosage is high; this is because of with the increase of RAP dosage, the proportion of new aggregate added to the mixture is also decreasing. Under the long-term load and aging effect of RAP, the strongest material is lower than that of new aggregate, which cannot well resist the vertical load, the vertical deformation increases, and the compressive resilient modulus shows a downward trend. Although the strength and stiffness loss of PCRM after the incorporation of RAP is large, the unconfined compressive strength is large, and the compressive resilience modulus of PCRM is maintained between 2700~3500 MPa, which is greater than that of the same grade of asphalt mixture (1500 Mpa). Therefore, PCRM has the characteristics of high strength and high modulus.

### 4.4. Low-Temperature Crack Resistance

Combined with the characteristics of polyurethane binder, the low-temperature crack resistance of PCRM was evaluated by low-temperature beam-bending test. The fully solidified rut plate specimen was cut into a 250 mm × 30 mm × 35 mm trabecular cuboid specimen. Before the test, the trabecular cuboid specimen was placed in a thermostatic refrigerator at minus 10 °C. When the internal temperature of the specimen reached the requirement, it was immediately placed on the support with a span of 200 mm for the test. The test machine was loaded at a loading rate of 50 mm/min until the specimen was destroyed. According to the test results, the relationship curves of PRCM flexural strength and maximum flexural train with RAP dosage were drawn, as shown in Figure 9c.

It can be seen from Figure 9c that with the increase in RAP dosage, the maximum flexural tensile strain and flexural tensile strength of the mixture decreased gradually. The higher the RAP dosage, the faster the maximum flexural tensile strain of the mixture decreased. The minimum value of maximum bending tensile strain of PRCM with five RAP dosages is 26,594 με, which is 7.5 times that of SBS-modified asphalt mixture (about 3500 με). The test results show that the low-temperature flexibility of polyurethane binder after curing is excellent. Asphalt aging is the main reason for the low-temperature shrinkage cracking of pavement; with the increase of RAP dosage, the content of aged asphalt increases, the toughness decreases at low temperature, and the overall performance of the mixture decreases, resulting in the decrease of the ability of PCRM to resist deformation at low temperature, which makes it easier to crack.

The low-temperature crack resistance of PCRM was evaluated by the maximum bending strain. The results show that the maximum bending strain of the mixture at various dosages maintained a large value, and PCRM was not prone to cracking at low temperatures. The maximum bending strain is negatively correlated with the RAP content. Compared with the mixture with 0% RAP content, the maximum bending strain of PCRM with 20%, 40%, 60%, and 80% content decreased by 5.5%, 15.4%, 21.0%, and 25.7%, respectively. This is because RAP asphalt aging brittleness is larger, low temperature will lead to the toughness of the mixture being reduced, and the ability to resist damage decreased.

### 4.5. Microscopic Morphology of Mixture

**Figure 10:**
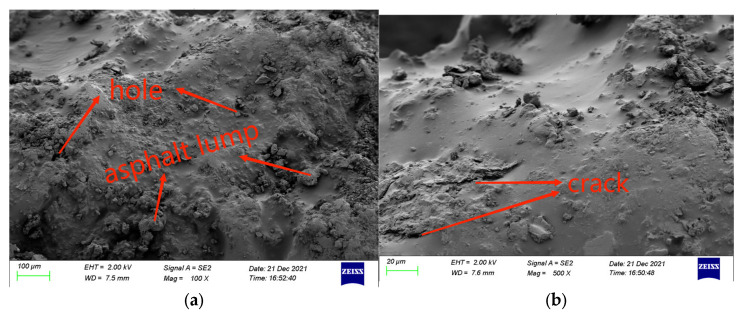
Microscopic morphology of PCRM mixture with 60% RAP dosage. (**a**) Amplified factor 100. (**b**) Amplified factor 500.

**Figure 11:**
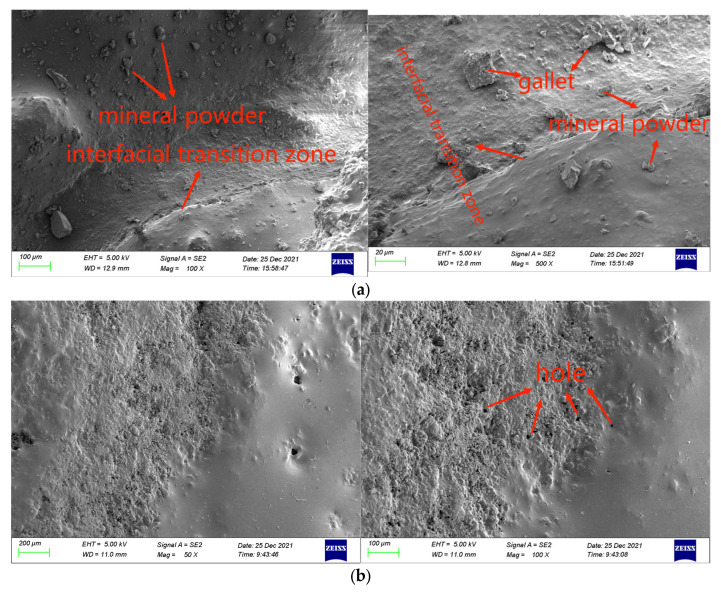

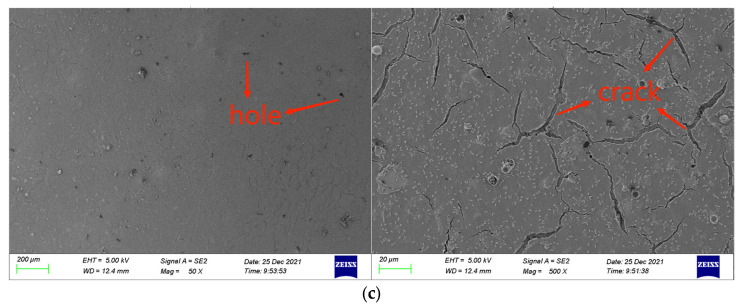
The microscopic morphology of PCRM under different dosages and environments. (**a**) Microscopic morphology of PCRM mixture with 0% RAP dosage. (**b**) Microscopic morphology of PCRM after immersion in water. (**c**) Microscopic morphology of PCRM after freezing and thawing.

To explore the influence of RAP on the internal structure of the mixture, the micro-morphology characteristics of the mixture with and without RAP were first analyzed. Figure 10 shows the micromorphology of PCRM with 60% RAP dosage, while Figure 11a shows the micromorphology of PCRM with 0% RAP dosage. Figure 11b shows the micromorphology of PCRM after immersion, and Figure 11c shows the micromorphology of PCRM after the freeze–thaw cycle.

From Figure 10 and Figure 11a, it can be seen that there are great differences between the flatness and smoothness of specimen surface in the microscopic morphology of the above two mixtures. It can be seen from Figure 10 that the surface condition of the sample after RAP incorporation is relatively complex; after amplification 100 times, it can be obviously seen that there are many holes and asphalt blocks on the surface of the sample. After amplification of 500 times, defects such as pores and cracks can be clearly seen on the surface of the sample; these micron-level defects and damage will have negative effects on the mechanics and durability of PCRM. Figure 11a shows that when RAP is not added in the mixture, the surface of the sample is relatively flat and smooth and the interface transition zone between polyurethane and aggregate can be obviously observed. From the interface transition zone, polyurethane can effectively fill the void between aggregates. From the microscopic morphology of the two mixtures amplified by 500 times, the polyurethane binder forms a smooth film on the surface of the old and new aggregates, showing that the new and old aggregates can be tightly wrapped by polyurethane, and that the adhesion performance is good, which is one of the reasons why the performance of the polyurethane mixture is better than that of asphalt mixture. In summary, due to the difference in microstructure on the surface of aggregates, the interface structure between polyurethane and new and old aggregates is greatly different.

From Figure 11b,c, we can observe the change in the microscopic morphology of the cold polyurethane cold recycling mixture under the action of water temperature. The scanning electron microscope images of PCRM samples under different water temperature environments are quite different. It can be seen from Figure 11b that under the immersion condition after the sample was enlarged 50 times, the aggregate part was exposed, and the polyurethane binder was spalled from the aggregate surface. There are large holes in the un-spalled part, which may be because the RAP surface asphalt reaches its softening point at high temperature and enters the sample after having a certain fluidity. After the exposed part of aggregate was amplified by 100 times, many holes were observed in the sample, indicating that the polyurethane binder began to spall and gradually spread from the holes on the RAP surface. It can be seen from Figure 11c that in the freeze–thaw state, after amplification of the sample by 50 times, it is found that the aggregate did not appear as naked as after immersion; after amplification by 500 times, although the binder on the surface of the sample did not completely peel off, a large number of micro-sized cracks were produced, and it is likely to completely spall after multiple freeze–thaw cycles. The microscopic morphology under these two test conditions shows that after the mixture is subjected to the alternating action of low temperature and high temperature, the rheological property of the binder is poor, and internal damage can easily occur, resulting in the insufficient water stability property of the mixture.

The microstructure of PCRM with RAP and without RAP was compared and analyzed by SEM. It was found that the surface of the two mixtures was covered with a relatively uniform polyurethane binder film. After the incorporation of RAP, due to the difference in the microstructure of the RAP surface, the polyurethane–aggregate interface structure was greatly different. The surface of the mixture was rougher than that of the mixture without RAP, and there were many micron-scale pores, cracks, and other defects on the surface. These micron-scale defect damages will negatively affect the mechanics and durability of PCRM.

### 4.6. Void Structure

Different void structures in the mixture will cause differences in the road performance of the mixture. The number and size of voids are closely related to the road performance of PCRM. With the continuous progress of industrial CT scanning technology, the internal information of the mixture can be accurately obtained. At the same time, using image processing software, the internal defects of the mixture can be judged, and its microscopic characteristics can be explored.

**Figure 12:**
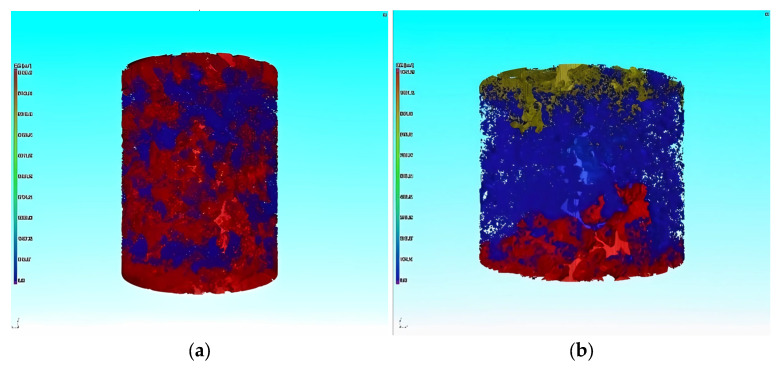
Three-dimensional void images of mixture under different molding methods. (**a**) Vibratory compaction. (**b**) Marshall compaction molding.

Application of reference industrial CT in asphalt mixture. Firstly, the mixture was scanned by industrial CT, and then the PCRM internal image was imported into VG Studio MAX software (hereinafter referred to as VGS) of Volume Graphics for three-dimensional structure processing; the software can obtain the three-dimensional image of the whole mixture. Next, using the “defect expansion” module in the complementing software, the maximum and minimum void volume to be calculated were set to 600 mm^3^ and 0 mm^3^, respectively, and then the three-dimensional void volume and number in the mixture were calculated, and the volume, number, and void distribution of the void in the mixture were analyzed by the information output by VGS software. Figure 12 is the three-dimensional gap image of PCRM extracted by VGS software under different molding methods.

**Figure 13:**
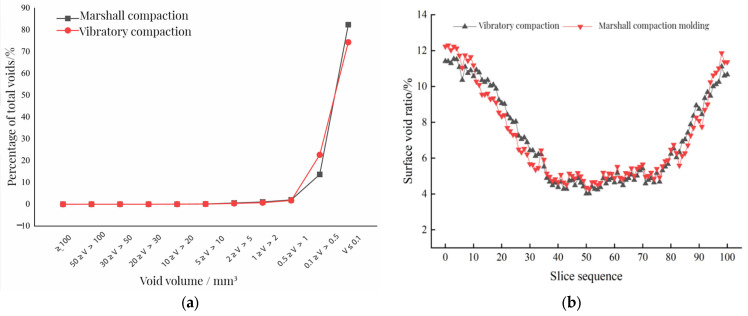
The void gradation and surface porosity of PCRM under different molding methods. (**a**) PCRM void gradation distribution. (**b**) Void distribution characteristics of different forming methods.

Firstly, the void gradation of the mixture is studied. Void gradation refers to the percentage of the volume void number and the total void number in the PCRM specimen. The void information was output to the Excel (2016, Microsoft, Albuquerque, NM, USA) table by VGS, then the total void number and the volume void number were statistically analyzed to obtain the void gradation of PCRM under different molding methods. The statistical results are shown in Figure 13a.

To study the longitudinal void distribution of PCRM under different molding methods, this section introduces the surface porosity to evaluate the void distribution along the specimen height. The surface porosity is the ratio of void area to section area in each fault image. Firstly, the VGS software was used to divide the three-dimensional reconstruction specimens of two kinds of mixtures into 100 continuous cross-section images evenly along the height. Then, after Image-J processing, the void area parameters were extracted, and the void rates of the cross-sections at different heights of the mixture were counted. The Excel chart was imported to calculate the ratio of void area to cross-section area in each fault image. Finally, the distribution of the surface porosity on the PCRM surface was statistically analyzed, and the relationship between surface porosity and specimen height is shown in Figure 13b.

Figure 13a shows that the ratio of large voids to total voids in PCRM produced by the two molding methods is close to 0%, and more than 95% of the voids are smaller than 1 mm^3^. The influence of different molding methods on the distribution of internal voids in PCRM was mainly concentrated in the voids with a void volume less than 1 mm^3^; the voids with a void volume less than 0.1 mm^3^ in the PCRM formed by vibration compaction accounted for 80% of the total void volume, which was about 10% higher than that of the PCRM formed by Marshall compaction, and the void volume between 0.1 mm^3^ and 0.5 mm^3^ was about 10% lower than that of the PCRM formed by Marshall compaction. In summary, there are fewer pores with large void areas in PCRMs of the two molding methods, and it can be seen from the three-dimensional image of Figure 12 that large pores are mainly concentrated in the upper and lower parts of the specimen.

It can be seen from Figure 13b that the surface void ratio of the specimen fluctuates greatly along the longitudinal direction of the specimen, and the surface void ratio at both ends is large, which decreases rapidly near the middle of the specimen. At the same time, the porosity of the middle surface of the specimen is more evenly distributed than that of the two ends. Overall, the porosity is distributed along the height direction of the specimen, which is large at both ends and small in the middle.

**Figure 14:**
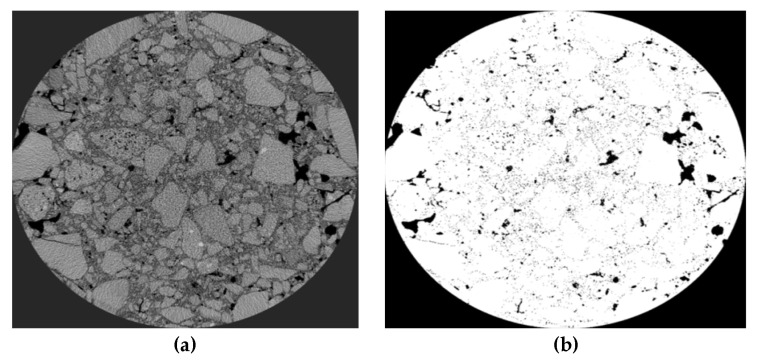
Section image processed by software. (**a**) Gray-scale image. (**b**) Space image after processing.

The void gradation obtained by VGS software only obtained the volume size and quantity distribution of internal voids in PCRM to explore the specific distribution law of void fraction along the whole height of the specimen. Firstly, the continuous cross-section images of the specimen after CT scanning were gray-processed by Matlab software (version 2020b), which is a commercial math software from MathWorks (Natick, MA, USA). Figure 14a shows the gray image obtained from the cross-section image after processing. Then, the gray image was threshold segmented by Image-J software. After adjusting the contrast, brightness, and gamma value, the void information of each cross-section image can be obtained, and after batch processing in this way, the distribution characteristics of the longitudinal void of the specimen can be accurately analyzed. The fault images before and after extraction were processed by the software as shown in Figure 14b.

In summary, the porosity of PCRM with two molding methods is small in the middle of the specimen, which is in a uniform state. This is because the aggregate of the specimen is relatively loose at the beginning of molding, and there are more coarse aggregates at the end of the specimen and the distribution is relatively stable. With the compaction process, the mold limits the movement of the aggregate on both sides, the coarse aggregate at the outer edge first forms a skeleton, and the freedom of the aggregate in the middle of the specimen is high, the contact between coarse and fine aggregates moves, and the fine aggregate gradually fills the gap, so that the specimen obtains better compactness in the middle. At the same time, the porosity of the specimen formed by vibration compaction is smaller than that of the specimen formed by Marshall compaction, because the Marshall compaction essentially imposes load on the mixture in the vertical direction by the drop hammer. At the same time, under the limitation of the mold, the aggregate falls vertically to squeeze and compact each other. The vibration molding produces a vibration pressure wave in the mixture during the compaction process, which reduces the static friction force between aggregates. The aggregate changes from the initial static state to the motion state, so that the aggregate is rearranged, and a more stable and dense void structure is obtained.

### 4.7. Failure Mechanism

**Figure 15:**
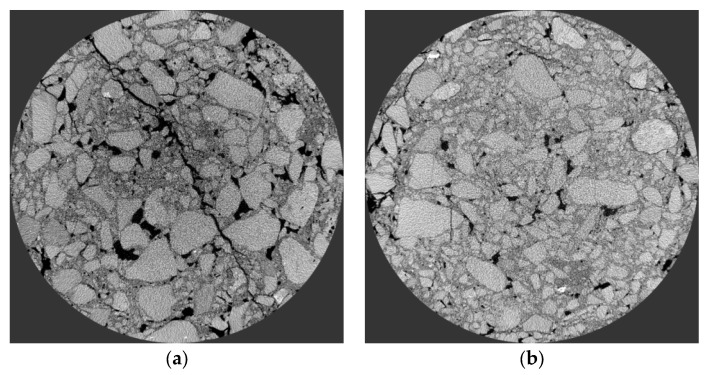
Fracture scanning maps under different loads. (**a**) After splitting test. (**b**) After uniaxial compression failure.

To explore the distribution characteristics of voids and cracks in the mixture under different stress modes, the PCRM specimens after splitting failure and uniaxial compression failure underwent industrial CT for scanning. The CT images of the middle layer of the specimen were selected, and the failure mode of PCRM was analyzed by intuitive analysis. The CT scan images of the middle layer of the specimen are shown in Figure 15.

It can be seen from Figure 15 that the internal crack formed in PCRM specimens after failure under different loads. After the splitting failure of PCRM, there was a crack through the whole specimen in the middle of the specimen, and there was a small crack propagation nearby. However, many cracks were generated at the edge of PCRM after uniaxial compression, but no obvious cracks were found in the specimen. This qualitative analysis method cannot effectively judge the failure degree of the specimen. To quantitatively analyze the damage degree of PCRM under different loads, Image J software (2019, National Institutes of Health, Bethesda, MD, USA) was used to process the tomographic images. The void area of the fault image before the specimen failure and the void and crack area of the fault image after the specimen failure were extracted, and then the increment of the void and crack before and after the failure was calculated. Finally, the failure coefficient was introduced to quantitatively analyze the damage degree of PCRM. The calculation formula of failure coefficient F is shown in Formula (1).

(1) $$F=\frac{S_{a}-S_{b}}{S_{b}}\times 100\%$$

In the formula: F—failure coefficient. $S_{a}$—The void and crack area of the fault image after specimen failure (mm^2^). $S_{b}$—The void area of the fault image before specimen failure (mm^2^). 

**Figure 16:**
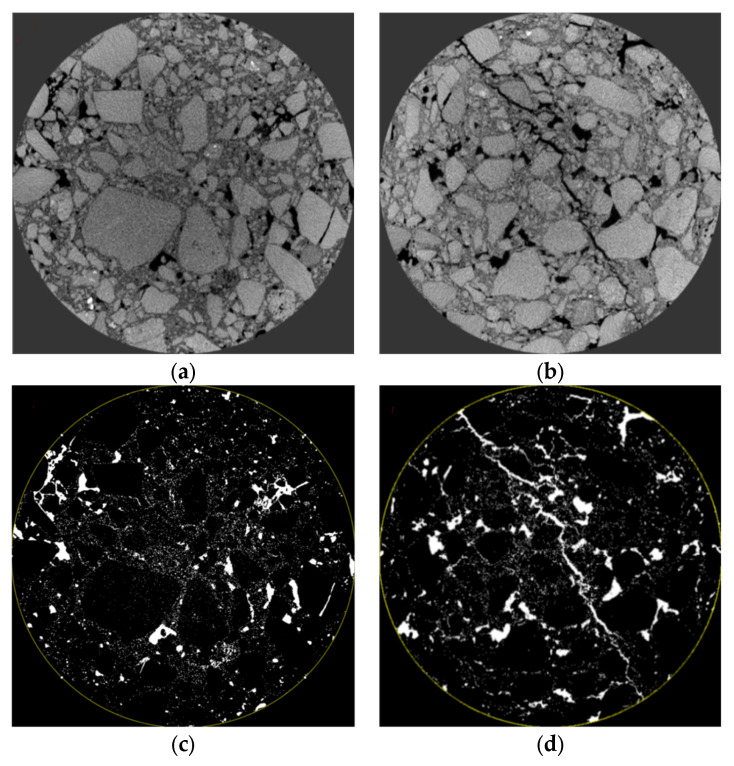
Distribution of void and crack before and after upper splitting of specimen. (**a**) Section image before destruction. (**b**) Section image after destruction. (**c**) Images of voids and cracks before failure. (**d**) Images of voids and cracks after failure.

The scanning maps of the upper, middle, and lower layers before and after the failure of specimens under two failure modes were selected for comparative study. Figure 16 shows the upper fault images before and after the splitting failure of the specimen and the processed images by Image J software, where a represents the section image before the failure, b represents the broken section image, and c, d represent a, b through Image J processing gap and crack image.

It can be seen from Figure 16 that there are a small number of small cracks near the pressure head after the splitting failure of the specimen, and there are cracks throughout the specimen in the middle position of the specimen; this is because the specimen is in the early stage of load loading, the pressure head first produces huge compressive stress at the edge of the mixture, resulting in local plastic deformation of the specimen. With the increase of load, the crack at the edge of the specimen continues to absorb the local plastic deformation of the specimen, and the crack continues to diffuse, resulting in cracks throughout the whole specimen. Then, the “Measure” command of Image J is used to measure the void area ${\;S}_{b}$ before the splitting failure of each layer of the specimen and the void and crack area $S_{a}$ after the splitting failure of each layer of the specimen. The failure coefficient of each layer of the specimen under the condition of the splitting test can be obtained from Formula (1). The results are shown in Table 8:

It can be seen from Table 8 that the void and crack area of the upper and lower layers of the specimen before and after failure are significantly larger than those of the middle layer of the specimen; this is because the void ratio in the middle of the specimen is smaller than that at both ends of the specimen, but the failure coefficients of the specimen in the upper, middle, and lower layers are basically the same, indicating that under the splitting failure mode, the crack area and the increment of void change after failure are basically the same, and the damage degree of the specimen is basically the same. By calculating the average failure coefficient of the upper, middle, and lower layers of the specimen, it can be determined that the failure coefficient F of the specimen under splitting failure is 25.0%.

**Figure 17:**
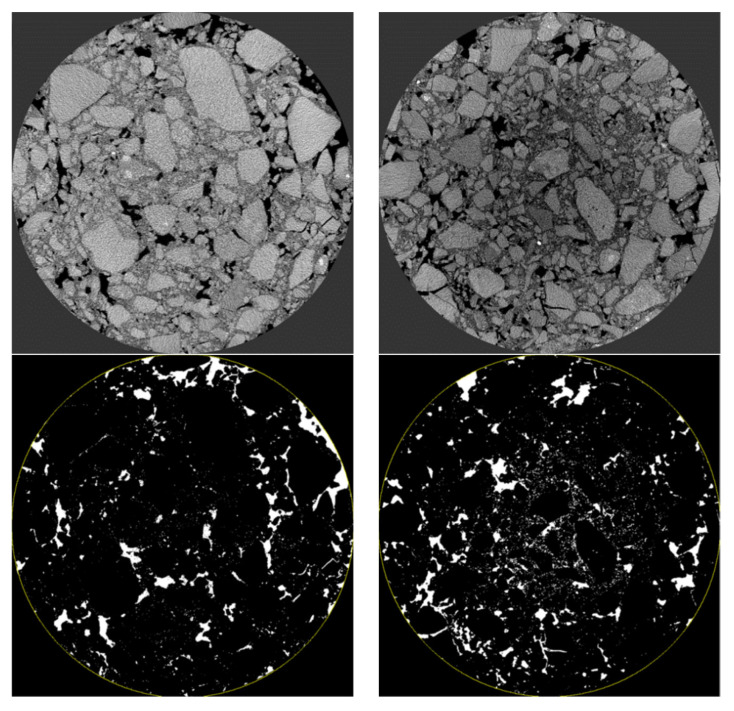
Distribution of void and crack before and after upper splitting of specimen.

**Figure 18:**
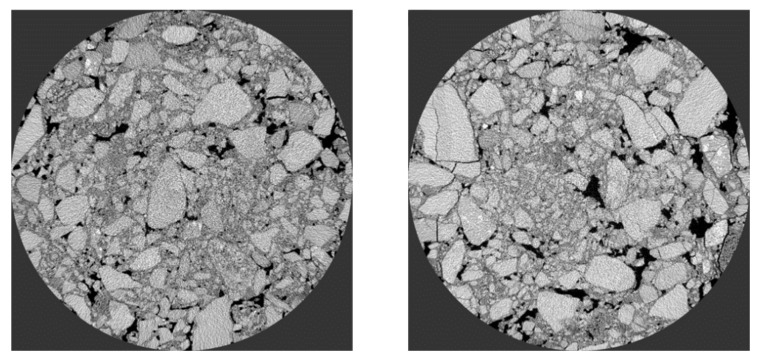

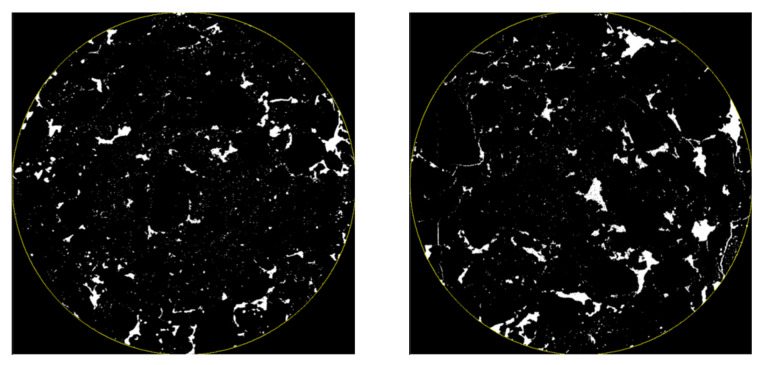
The distribution maps of voids and cracks before and after middle splitting of the specimen.

**Figure 19:**
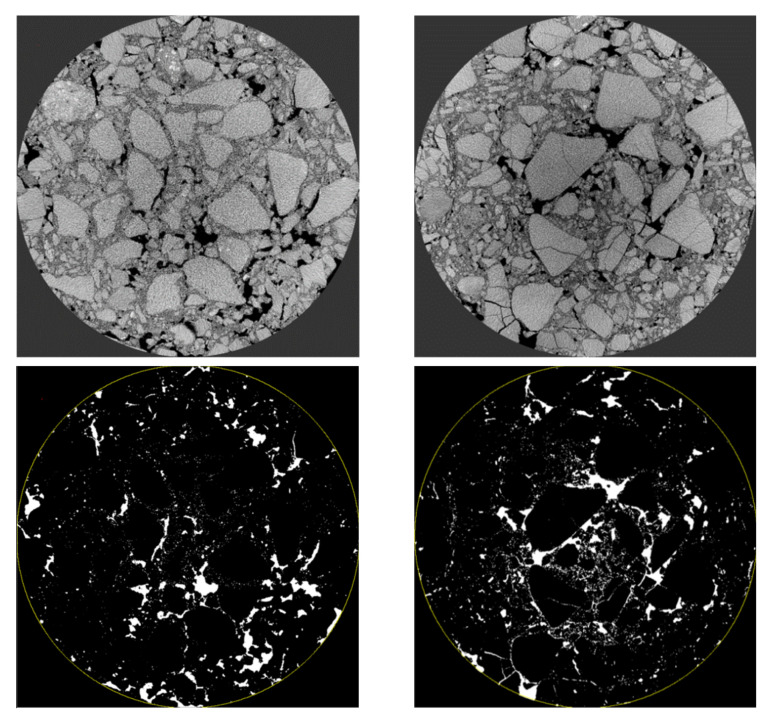
The distribution of void and crack before and after splitting of lower specimen.

Similarly, the specimens before and after uniaxial failure were placed in industrial CT for scanning. After the scanning, the typical fault images in the upper, middle, and lower layers were selected, and the images were processed by Image J to extract the void and crack area in the images. The section images of the specimens in the upper, middle, and lower layers and the processed images are shown in Figure 17, Figure 18 and Figure 19. 

It can be seen from Figure 17, Figure 18 and Figure 19 that a fine crack appeared on the aggregate surface of the upper and lower layers of the specimen before uniaxial failure, which was due to the weak ability of RAP to resist vertical load and the internal fracture during molding. However, the aggregate in the middle layer of the specimen was not damaged, the inter-aggregate was compacted, and the porosity was small. Observing the CT image of the innermost part of the specimen, it is not found that there is a crack through the specimen after splitting; the development of the crack is mainly concentrated in the edge part of the asphalt mixture specimen and the surface of the aggregate. The cracks on the surface of the aggregate after failure are significantly increased compared with those before failure.

The void and crack area of specimens before and after failure were extracted by Image J, and the failure coefficient was calculated. The calculation results are shown in Table 9.

From Table 9, the failure coefficient of the middle layer of the specimen after uniaxial compression failure is significantly greater than that of the upper and lower layers of the specimen. This is because the upper and lower layers of the specimen are constrained by the lateral constraints of the pressure head, which limits its deformation; the lateral strain of the middle layer of the specimen has been continuously developed, resulting in obvious uplift in the middle layer of the specimen and more serious damage. Through the continuous pressure on the specimen, the phenomenon of uplift in the middle of the specimen can be seen. After taking the average value of the failure coefficient of the upper, middle, and lower layers of the specimen after uniaxial compression, the failure coefficient F of the PCRM specimen under the uniaxial compression test is 22.5%. By comparing the failure coefficients of specimens with splitting failure mode and the uniaxial failure mode, the failure coefficient of specimens under the uniaxial compression test is smaller than that under the splitting test. After the splitting test, huge cracks were running through the whole specimen in the middle of the specimen, and the area of cracks was large, but no obvious cracks appeared in other parts of the specimen. Under uniaxial test conditions, cracks mainly occur at the edge of the specimen and the surface of the aggregate; although there are cracks inside the specimen, these cracks are all small cracks.

The internal structure of PCRM was analyzed by industrial CT three-dimensional imaging technology. It was found that the voids of PCRM formed by vibration compaction and Marshall compaction were mainly distributed below 1 mm^3^, and vibration compaction could improve the compactability and structural uniformity of PCRM compared with Marshall compaction. In the distribution of surface porosity along the height of the specimen, the porosity at both ends of the specimen is large, the porosity in the middle of the specimen is small, and the distribution is uniform.

Although some achievements have been made in this article, there are still many shortcomings in the research work of this paper. The following problems need to be further solved:

(1) In this paper, only the PCRM of continuous dense gradation is studied. It is suggested that the cold-recycled mixture of open gradation and gap gradation should be studied to better promote and use new materials.
(2) This paper does not carry out long-term road performance research. It is recommended to lay test roads and observe the fatigue resistance, aging resistance, and chemical corrosion resistance of PCRM during long-term service.

## 5. Conclusions

(1) By analyzing the road performance of PCRM with different RAP dosages, it was found that due to the low sensitivity of polyurethane binder to temperature, PCRM showed better high- and low-temperature performance than asphalt mixture, with high strength and high modulus. However, under the coupling effect of water temperature, the water stability of PCRM was poor. Because of the instability of RAP material, the dosage of RAP has a great influence on the road performance of the mixture.
(2) Through the adhesion test found that polyurethane binder and natural aggregate and RAP have good adhesion properties, the range of the binder ratio of PCRM was between 8% and 10%. High temperature helps to accelerate the formation strength of the mixture, but the maintenance conditions do not affect the final strength of the mixture. In practical use, PCRM can be maintained at maximum strength at room temperature after high-temperature-accelerated maintenance. With the dry–wet splitting strength ratio as the design index, the splitting strength of PCRM with four RAP dosages increased greatly when the glue ratio reached 9%, the dry–wet splitting strength ratio could meet the requirements of the regeneration specification, and the optimal binder ratio was finally determined to be 9%.
(3) After the incorporation of RAP, the microscopic morphology of PCRM was rougher than that without RAP, and there were a large number of micron-scale defects, such as holes and cracks, which will have a negative impact on the mechanical and durability of PCRM. After soaking in water, the polyurethane peeled off at the holes on the surface of RAP. Under the freeze–thaw condition, the polyurethane film on the surface of PCRM produced a large number of cracks, which may spall after repeated freeze–thaw cycles, further verifying the conclusion that PCRM has poor water stability in macroscopic road performance.
(4) Pores with a pore volume less than 1 mm^3^ in PCRM accounted for more than 95% of the total pore volume under vibratory compaction and Marshall compaction. By comparing the surface porosity of PCRM along the height direction of the specimen, it was found that the void distribution of the vibration compaction specimen was relatively uniform, and the porosity in the longitudinal distribution of the specimen under the two molding methods presents the phenomenon of “large at both ends and small in the mid”. Under different loads, there were large penetrating cracks in the middle of the specimen after splitting failure, and there were a large number of small cracks on the edge and aggregate surface after uniaxial failure. The void and crack area of specimens before and after failure were extracted by Image J software, and the failure degree of the mixture was quantitatively analyzed by the failure coefficient; the failure coefficient was used to quantitatively analyze the failure degree of the mixture, and it was found that the failure degree of the mixture after splitting test was greater than that of a uniaxial compression test.

## Figures and Tables

**Table 1:** Gradation after RAP classification.

Fraction	Percentage of Passing through Each Sieve Aperture (mm) (%)											
	26.5	19	16	13.2	9.5	4.75	2.36	1.18	0.6	0.3	0.15	0.075
0~5 mm	100	100	100	100	100	60.2	45.0	33.3	22.3	13.7	13.1	4.8
5~10 mm	100	100	100	100	97.9	2.2	0.6	0	0	0	0	0
10~20 mm	100	78.1	55.9	29.3	4.8	1.7	0	0	0	0	0	0

**Table 2:** Test conclusion of recycled asphalt performance.

Asphalt Type	25 °C Penetration Degree (0.1 mm)	Softening Point (°C)	15 °C Ductility (mm)	135 °C Kinematic Viscosity (Pa·s)
asphalt	74.6	48.5	>149.2	1.22
recycled asphalt	14.6	58.9	46	3.34
technical standard	60~80	≮46	≮100	-

**Table 3:** Test results on physical properties of natural aggregates.

Index	Apparent Density (g/cm^3^)	Hygroscopic Rate (%)	Crush Value (%)	Flat Elongated Particle Content (%)	Sand Equivalent (%)	Los Angeles Wear Value (%)
10–20 mm	2.674	0.42	12.8	8.4	2.652	18.5
5–10 mm	2.669	0.56	-	9.6	0.68	17.2
0–5 mm	2.652	0.68	-	-	70	-
filler	2.670	-	-	-	-	-

**Table 4:** Natural aggregate screening test results.

Material Type	Percentage of Passing through Each Sieve Aperture (mm) (%)											
	26.5	19	16	13.2	9.5	4.75	2.36	1.18	0.6	0.3	0.15	0.075
0~5 mm	100	100	100	100	100	99.6	31.9	24.3	17.0	13.1	7.4	4.2
5~10 mm	100	100	100	100	98.5	5.6	0	0	0	0	0	0
10~20 mm	100	95.3	93.2	39.3	1.2	0.1	0	0	0	0	0	0

**Table 5:** Properties of polyurethane adhesive.

Test Item	Density (g/cm^3^)	Viscosity (mPa·s)	Surface Dry Time (h)	Hard Drying Time (h)	Tensile Strength (MPa)	Elongation at Break (%)
test result	1.1	3400	1.5	8	10	323
test method	GB4472	GB2794	GB13477	GB16777	GB16777	/

**Table 6:** Results of PCRM dry–wet splitting strength test with four kinds of RAP dosage.

Reclaimed Materials Dosage (%)	Binder Ratio (%)	Splitting Strength at 15 °C (MPa)	Splitting Strength after 24 h Immersion (MPa)	Dry–Wet Splitting Strength Ratio (%)
20	8	3.33	2.45	72.8
	9	3.67	2.81	76.3
	10	3.75	3.18	84.6
40	8	3.41	2.44	71.4
	9	3.78	2.99	79.2
	10	3.82	3.14	82.1
60	8	3.26	2.37	72.6
	9	3.54	2.70	76.4
	10	3.64	2.95	81.2
80	8	3.14	2.27	72.3
	9	3.42	2.68	78.4
	10	3.50	2.89	82.6

**Table 7:** Experimental results of high-temperature stability.

RAP Dosage (%)	Stability (KN)	Flow Value (mm)	60 min Rut Depth (µm)	Dynamic Stability (Times/mm)
0	47.4	28.4	620	35,342
20	44.4	27.3	690	33,287
40	43.1	25.4	730	32,340
60	41.2	26.4	770	30,297
80	39.4	32.8	840	27,684

**Table 8:** Failure coefficient of upper, middle, and lower layers after splitting failure.

Layer	Void Area before Destruction (mm^2^)	Void, Crack Area after Failure (mm^2^)	Coefficient of Destruction (%)	Average Value (%)
upper	562.341	764.252	26.0	25.0
middle	464.587	621.558	25.3	
lower	577.450	755.731	23.6	

**Table 9:** Failure coefficient of upper, middle, and lower layers before and after uniaxial failure.

Layer	Void Area before Destruction (mm^2^)	Void, Crack Area after Failure (mm^2^)	Coefficient of Destruction (%)	Average Value (%)
upper	542.742	661.583	18.0	22.5
middle	334.953	507.705	34.0	
lower	518.647	613.269	15.4	

## Data Availability

All data that support the findings of this study are included within the article.
